# Association of overactive bladder with all‐cause and cardiovascular mortality in women: A propensity‐matched NHANES study

**DOI:** 10.1002/bco2.70022

**Published:** 2025-04-29

**Authors:** Weipu Mao, Sagar Barge, Zhaobo Luo, Weiqun Yu

**Affiliations:** ^1^ Department of Medicine, Beth Israel Deaconess Medical Center Harvard Medical School Boston Massachusetts USA

**Keywords:** all‐cause mortality, cardiovascular mortality, overactive bladder, propensity score matching, triglyceride‐glucose index

## Abstract

**Objectives:**

To examine the impact of overactive bladder (OAB) on all‐cause and cardiovascular mortality in women in a real‐world setting, and to examine the association of TyG‐related indices with OAB.

**Methods:**

Data on 6580 women aged ≥20 years were collected from the National Health and Nutrition Examination Survey (NHANES) database. Kaplan–Meier curves and Cox survival analysis were used to evaluate the association between OAB and all‐cause and cardiovascular mortality. Biomarkers for metabolic syndrome were assessed for their association with OAB, including triglyceride‐glucose (TyG) and TyG‐related indices. The association between TyG‐related indices and OAB was evaluated using restricted cubic splines (RCS), receiver operating characteristic (ROC) curves and multivariate logistic regression, with propensity score matching (PSM) employed to balance confounders between OAB and non‐OAB groups.

**Results:**

Kaplan–Meier curves showed that OAB was associated with a poorer prognosis, and multivariate Cox regression analyses indicated that OAB was an independent risk factor for both all‐cause and cardiovascular mortality. RCS revealed a positive association between TyG‐related indices and OAB. Both ROC curves and multivariate logistic regression analysis indicated that TyG‐WHtR (TyG combined with waist‐to‐height ratio) was strongly associated with OAB, with a higher TyG‐WHtR associated with an increased risk of OAB. The retrospective design and selection bias may be the potential limitations.

**Conclusions:**

OAB is positively associated with all‐cause and cardiovascular mortality in women. TyG‐related indices are positively associated with OAB, with TyG‐WHtR as the most effective index.

**Abbreviations**
OAB: overactive bladder
TyG: triglyceride‐glucose index
BMI: body mass index
WC: waist circumference
TyG‐BMI: TyG combined with BMI
TyG‐WC: TyG combined with WC
TyG‐WHtR: TyG combined with waist‐to‐height ratio
NHANES: National Health and Nutrition Examination Survey
RCS: Restricted cubic spline
ROC: Receiver operating characteristic
PSM: Propensity score matching
IR: Insulin resistance
TG: triglycerides
FBG: fasting blood glucose
NCHS: National Center for Health Statistics
OABSS: overactive bladder symptom score
HDL: high‐density lipoprotein
LDL: low‐density lipoprotein
TC: total cholesterol
NDI: National Death Index
KM: Kaplan–Meier; HR, hazard ratios
CI: confidence intervals
OR: odds ratios
AUC: area under the curve
TVD: three‐vessel disease

## 1 INTRODUCTION

OAB is clinically characterized by urgency, increased frequency, nocturia and urinary incontinence.^1^ OAB afflicts over 17% of the US population^2^ and approximately 20.8% of the people in Asia.^3^ The prevalence of OAB varies by sex, with women being more likely to develop OAB than men. For example, OAB affects 10.8% of men and 12.8% of women in Europe and Canada.^4^ OAB significantly impacts the quality of life of patients, and severe OAB in female patients can serve as an independent risk factor for bone fractures.^5^, ^6^ However, the impact of OAB on overall survival has not yet been reported.

Many factors could contribute to OAB, such as infection, injuries, outlet obstruction and ageing. Among these, diabetes is the most common significant risk factor for OAB. Diabetes affects approximately 463 million people worldwide, and this number is expected to rise continuously to around 700 million by 2045.^7^, ^8^ Diabetes mellitus (DM) results in a range of complications,^9^ including the prevalent diabetic bladder dysfunction (DBD), which often accounts for 40%–100% of DM patients. DBD is characterized by symptoms of overactive bladder (OAB), particularly in the early stage of DM.

Insulin resistance (IR) is the cornerstone of type 2 diabetes, characterized by reduced insulin secretion or inadequate sensitivity to insulin. The gold standard for assessing IR is the hyperinsulinemic‐euglycemic glucose clamp test.^10^ However, its complexity, time consumption and high cost prevent its wide implementation. Because IR leads to metabolic syndrome by abnormal energy and lipid metabolism,^11^ the triglyceride‐glucose (TyG) index has recently been widely used as a surrogate marker for IR. TyG is calculated using triglycerides (TG) and fasting blood glucose (FBG).^11^ Although the conventional TyG index has been widely adopted, its predominant reliance on lipid and glycemic parameters may inadequately reflect the comprehensive influence of obesity and fat distribution. Additional biomarkers derived from TyG, such as triglyceride‐glucose‐waist circumference (TyG‐WC), triglyceride‐glucose‐body mass index (TyG‐BMI) and triglyceride‐glucose‐waist‐to‐height ratio (TyG‐WHtR) have also been developed to study the occurrence and progression of diseases associated with metabolic syndrome and diabetes.^12^ TyG‐WC integrates waist circumference to assess visceral fat distribution, offering a more direct association between abdominal obesity and visceral adipose‐related IR.^13^ TyG‐BMI incorporates BMI to evaluate the metabolic impact of systemic obesity,^14^ while TyG‐WHtR employs waist‐to‐height ratio to standardize fat distribution assessment across individuals of varying heights.^15^

Diabetes, obesity and metabolic syndrome are well‐known risk factors for OAB. Recently, we reported that insulin signalling and adiponectin signalling play important roles in regulating bladder function, and disruption of these pathways leads to bladder dysfunctions like overactivity.^16^, ^17^ These signalling pathways are critical in energy and lipid metabolisms, including blood glucose and TyG. However, the association of TyG and its derived indices with OAB is unknown. We thus speculate that TyG and related indices might be strongly associated with OAB, and we will further evaluate whether OAB could impact all‐cause and cardiovascular mortality in women.

## 2 MATERIALS AND METHODS

### 2.1 Study population

The population included in this study was collected from the 2007 to 2018 National Health and Nutrition Examination Survey (NHANES). NHANES is a cross‐sectional study administered by the National Center for Health Statistics (NCHS) that assesses the health and nutritional status of the U.S. population. The survey collects demographic information, socioeconomic data, dietary details, physical examinations, laboratory tests and health‐related questions. The NHANES protocols were reviewed and approved by the NCHS Ethics Committee, and all participants signed informed consent forms during the survey process.^18^ The study included 30 213 female participants aged 20 years and older.

Exclusion criteria were as follows: a) unknown TyG‐related indices (n = 21 409); b) unknown OAB survey data (n = 2100); c) unknown education level and marital status (n = 8); d) unknown hypertension and diabetes status (n = 12); e) unknown physical activity status (n = 5); f) unknown smoking and alcohol consumption status (n = 8); g) unknown kidney function (n = 34); and (h) unknown blood lipid levels (n = 57). After excluding all relevant factors, a total of 6580 individuals were selected.

### 2.2 Study variables and outcome

The primary study variables include the diagnosis of OAB and the calculation of TyG‐related indices. The diagnosis of OAB was determined based on responses to the questions in the “Kidney Conditions ‐ Urology” section of the survey: KIQ044 (During the past 12 months, have you leaked or lost control of even a small amount of urine with an urge or pressure to urinate and you could not get to the toilet fast enough?), KIQ450 (How frequently does this occur?) and KIQ480 (During the past 30 days, how many times per night did you most typically get up to urinate, from the time you went to bed at night until the time you got up in the morning.). The Overactive Bladder Symptom Score (OABSS) was used to quantify the severity of OAB, with individuals having an OABSS ≥ three diagnosed as OAB.^19^ Specifically, the KIQ044 question was used to determine the presence or absence of urinary frequency, while KIQ450 and KIQ480 were used to quantify the OAB score. **Table** **S1** presents specific scoring criteria for quantifying OAB, based on symptom frequency conversion criteria recorded in NHANES and OABSS scores.

The calculation of the TyG‐related indices was as follows: TyG index = ln[TG (mg/dL) × FBG (mg/dL)/2], TyG‐BMI index = ln[TG (mg/dL) × FBG (mg/dL)/2] × BMI, TyG‐WC index = ln[TG (mg/dL) × FBG (mg/dL)/2] × WC and TyG‐WHtR index = ln[TG (mg/dL) × FBG (mg/dL)/2] × WC/Height.

Other covariates include age, race, educational level, marital status, hypertension, diabetes, smoking, alcohol consumption, participation in work activities, participation in recreational activities, BMI, WC, FBG, TG, high‐density lipoprotein (HDL), low‐density lipoprotein (LDL), total cholesterol (TC), blood urea nitrogen, creatinine and uric acid. Hypertension and diabetes are based on self‐reported physician‐diagnosis.

Individual mortality data were obtained from the NHANES public‐use linked mortality files through December 31, 2019, which link to the National Death Index (NDI) maintained by the NCHS (https://www.cdc.gov/nchs/data-linkage/mortality-public.htm#). All‐cause mortality refers to deaths from all causes, and cardiovascular mortality refers to deaths due to heart disease (054–068).

### 2.3 Statistical analysis

Categorical variables were presented as frequencies (n) and percentages (%) and were analysed using the Chi‐square test. A weighted analysis of the data was performed. Kaplan–Meier (KM) curves were used to evaluate the impact of the OAB on all‐cause and cardiovascular mortality. Multivariate Cox regression was employed to assess the association between OAB and all‐cause and cardiovascular mortality, reporting adjusted hazard ratios (aHR) with 95% confidence intervals (CI). To examine the association between TyG‐related indices and OAB, dose–response curves of restricted cubic spline (RCS) and logistic regression analysis were applied to calculate adjusted odds ratios (aOR) with 95% CI. Receiver Operating Characteristic (ROC) curves were used to evaluate the accuracy of TyG‐related indices for OAB, and the area under the curve (AUC) was reported.

We constructed four different models in Cox regression and logistic regression analyses. Model 1 is a univariate regression analysis; Model 2 adjusts for general covariates, including age, race, educational level and marital status; Model 3 further adjusts for disease conditions, including hypertension, diabetes, smoking and alcohol consumption; Model 4 adds additional adjustments for physical activity status, renal function and blood lipid levels. Propensity score matching (PSM) is a statistical method used to mitigate bias caused by non‐randomized group allocation in observational studies.^20^ To balance the differences between the OAB and non‐OAB groups, we used a 1:1 PSM method, adjusting for the following confounding variables: age, race, education level, marital status, hypertension, diabetes, smoking, alcohol consumption and physical activity status. Data were reanalysed after PSM to test the validity. All data and charts in this study were organized and analysed using R software (version 3.5.3) and SPSS software (version 25.0). A p‐value of less than 0.05 was considered statistically significant.

## 3 RESULTS

### 3.1 Demographic and clinicopathologic characteristics

Among the 6580 female participants, 1581 (24.0%) were diagnosed with OAB. **Table** **S2** presents the clinical baseline characteristics of the participants. The chi‐square test revealed significant differences between the OAB and non‐OAB groups in terms of age, race, educational level, marital status, hypertension, diabetes, smoking, alcohol consumption, moderate work activity and recreational activity (All p < 0.05). Patients with OAB were more likely to be older (>60 years, 49.7% vs. 26.2%), black (27.1% vs. 18.3%), less educated (high school: 33.3% vs. 19.7%), unmarried (60.7% vs. 51.0%), have hypertension (56.7% vs. 31.0%) and have diabetes (23.1% vs. 8.7%). Additionally, BMI, WC, FBG, TG, HDL, TC, TyG, TyG‐BMI, TyG‐WC, TyG‐WHtR, blood urea nitrogen, creatinine and uric acid levels were all significantly higher in the OAB group compared to the non‐OAB group.

### 3.2 OAB was strongly associated with all‐cause and cardiovascular death

We assessed the association of OAB with all‐cause and cardiovascular mortality among all participants using KM survival curves and Cox survival regression analyses. The KM survival curves showed that participants in the OAB group had higher all‐cause mortality (**Figure** **S1** **A**) and cardiovascular mortality (**Figure** **S1** **B**) compared to the non‐OAB group. Subsequently, the Cox survival regression analysis showed that OAB significantly increased the mortality risk for participants in all four models (Table 2 and **Table** **S3**). After adjusting for all variables (model 4), the OAB group had a 1.414‐fold (95% CI 1.167–1.714, p < 0.001; **Figure** **S2A**) increased risk of all‐cause mortality and a 1.528‐fold (95% CI 1.026–2.274, p = 0.037; **Figure** **S2B**) increased risk of cardiovascular mortality compared to the non‐OAB group.

**FIGURE 1:**
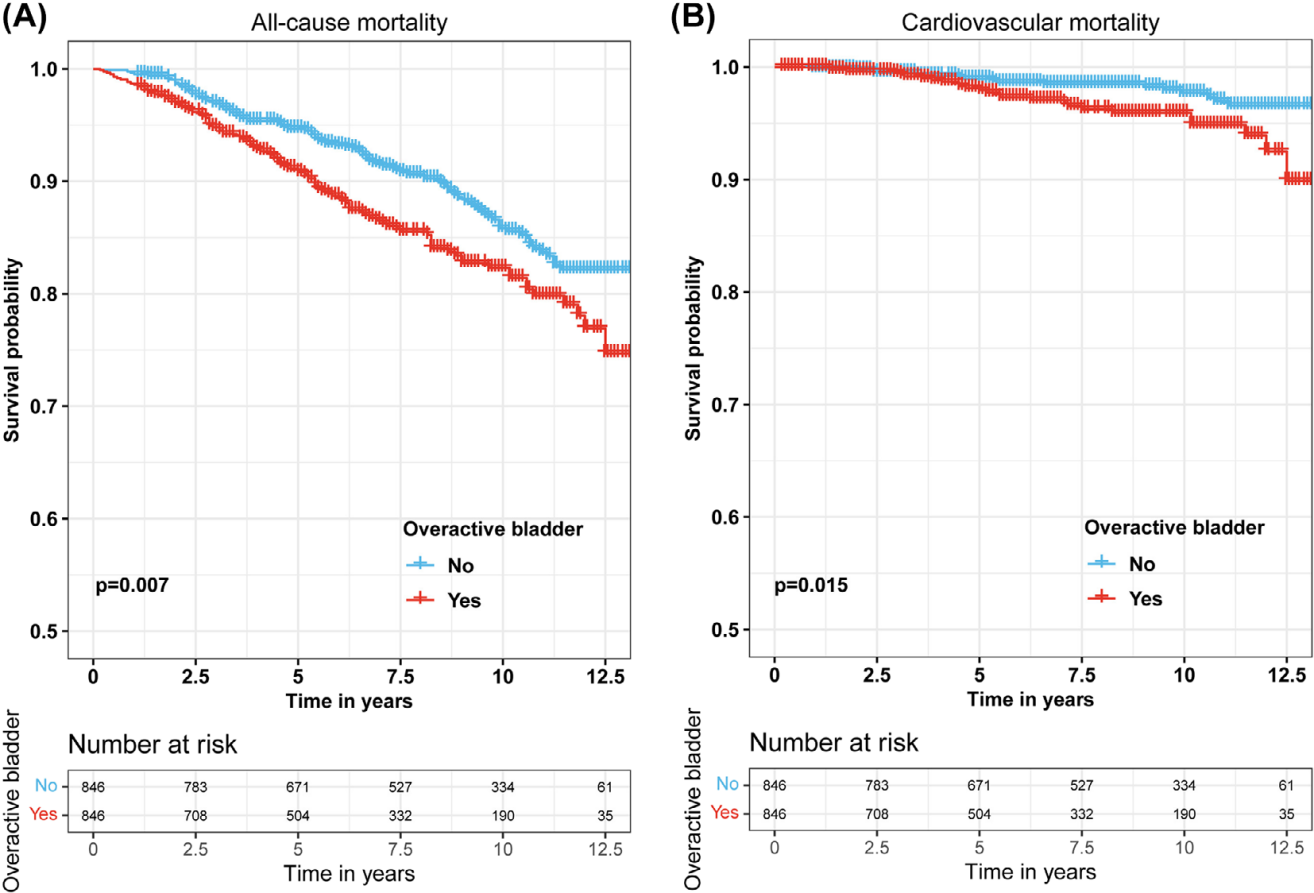
**All‐cause and cardiovascular mortality curves between the overactive bladder and the non‐overactive bladder group after 1:1 propensity score matching (PSM).** (A) All‐cause mortality curves; (B) cardiovascular mortality curves.

**FIGURE 2:**
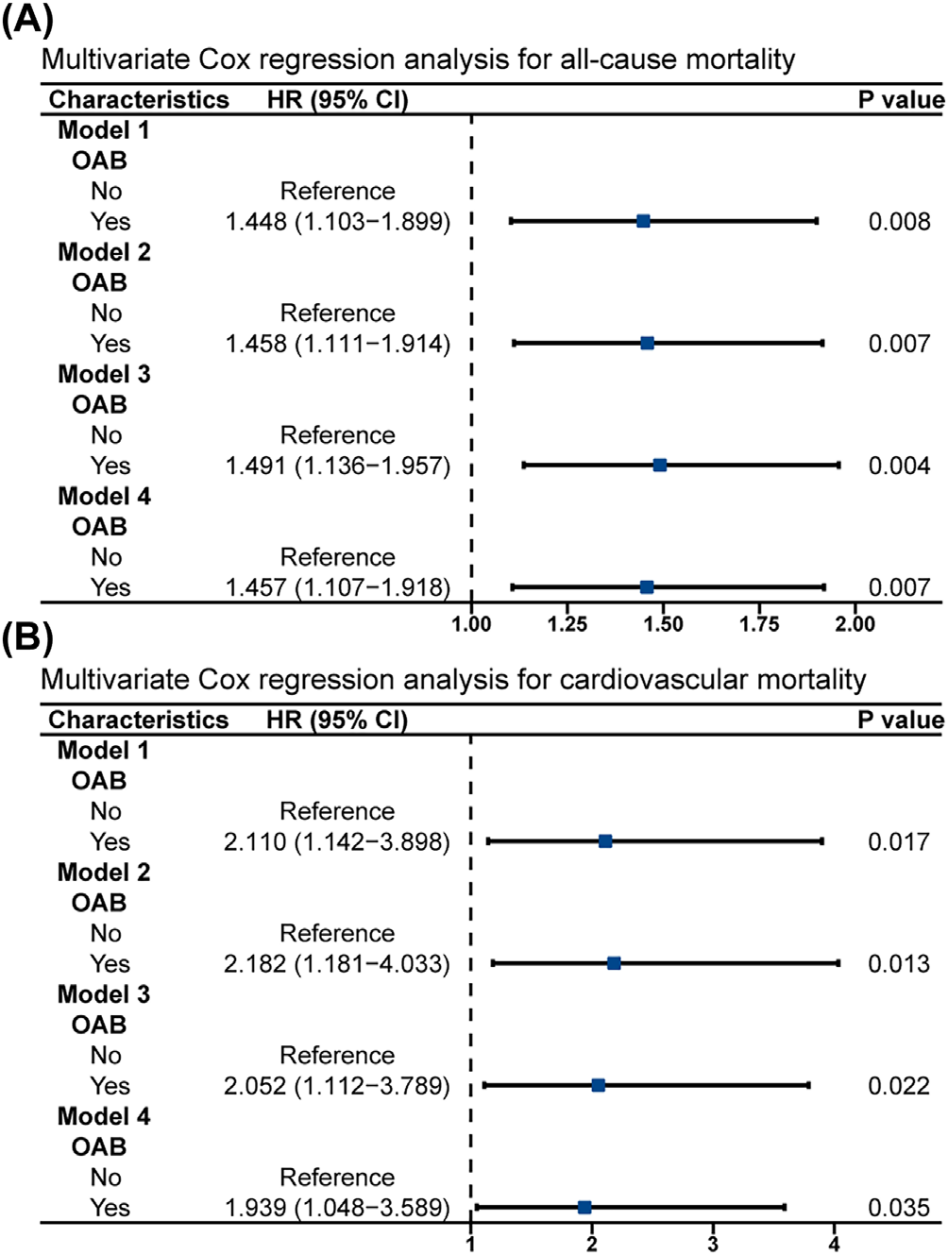
**Forest plots of different models of multivariate cox regression analysis of the relation between OAB and all‐cause and cardiovascular deaths in women after 1:1 propensity score matching (PSM).** (A) All‐cause mortality; (B) cardiovascular mortality.

To better balance the confounding factors between the OAB and non‐OAB groups, we performed a 1:1 PSM analysis, ultimately including 846 participants in both the OAB and non‐OAB groups. The clinical baseline characteristics of the 1692 participants after PSM analysis were presented in Table 1. After PSM, the OAB group still had higher BMI, WC, TyG‐BMI, TyG‐WC and TyG‐WHtR levels than the non‐OAB group. The KM survival curve results showed that the OAB group had higher all‐cause mortality (Figure 1A) and cardiovascular mortality (Figure 1B) compared to the non‐OAB group. Multivariate Cox regression analysis revealed a 1.457‐fold (95% CI 1.107–1.918, p = 0.007; Figure 2A) increased risk of all‐cause mortality and a 1.939‐fold (95% CI 1.048–3.589, p = 0.035; Figure 2B) increased risk of cardiovascular mortality in the OAB group compared to the non‐OAB group (Table 2 and **Table** **S4**).

**TABLE 1:** Baseline characteristics of participants between 2007 and 2018 after PSM.

Characteristics	Total	Non‐overactive bladder	Overactive bladder	*P* value
	No. (%)	No. (%)	No. (%)	
Total patients	1692	846 (50.0)	846 (50.0)	
Age, years				0.992
<40	344 (20.3)	171 (20.2)	173 (20.4)	
40–60	560 (33.1)	280 (33.1)	280 (33.1)	
>60	788 (46.6)	395 (46.7)	393 (46.5)	
Race				0.981
Mexican American	256 (15.1)	127 (15.0)	129 (15.2)	
Other Hispanic	165 (9.8)	81 (9.6)	84 (9.9)	
Non‐Hispanic white	773 (45.7)	393 (46.5)	380 (44.9)	
Non‐Hispanic black	393 (23.2)	193 (22.8)	200 (23.6)	
Other	105 (6.2)	52 (6.1)	53 (6.3)	
Education level				0.952
Less than high school	477 (28.2)	236 (27.9)	241 (28.5)	
High school or equivalent	350 (20.7)	177 (20.9)	173 (20.4)	
College or above	865 (51.1)	433 (51.2)	432 (51.1)	
Marital status				0.883
Married	713 (42.1)	355 (42.0)	358 (42.3)	
Unmarried	979 (57.9)	491 (58.0)	488 (57.7)	
Hypertension				0.846
Yes	802 (47.4)	403 (47.6)	399 (47.2)	
No	890 (52.6)	443 (52.4)	447 (52.8)	
Diabetes				0.846
Yes	211 (12.5)	102 (12.1)	109 (12.9)	
No	1464 (86.5)	736 (87.0)	728 (86.1)	
Borderline	17 (1.0)	8 (0.9)	9 (1.1)	
Alcohol consumption				0.764
Yes	1040 (61.5)	523 (61.8)	517 (61.1)	
No	652 (38.5)	323 (38.2)	329 (38.9)	
Smoking status				0.979
Never	1130 (66.8)	564 (66.7)	566 (66.9)	
Former	305 (18.0)	152 (18.0)	153 (18.1)	
Current	257 (15.2)	130 (15.4)	127 (15.0)	
Vigorous work activity				0.518
Yes	127 (7.5)	67 (7.9)	60 (7.1)	
No	1565 (92.5)	779 (92.1)	786 (92.9)	
Moderate work activity				0.643
Yes	386 (22.8)	197 (23.3)	189 (22.3)	
No	1306 (77.2)	649 (76.7)	657 (77.7)	
Vigorous recreational activity				0.766
Yes	109 (6.4)	56 (6.6)	53 (6.3)	
No	1583 (93.6)	790 (93.4)	793 (93.7)	
Moderate recreational activity				0.957
Yes	487 (28.8)	244 (28.8)	243 (28.7)	
No	1205 (71.2)	602 (71.2)	603 (71.3)	
BMI (kg/m^2^)	30.25 ± 7.35	29.17 ± 6.88	31.34 ± 7.64	<0.001
WC (cm)	100.05 ± 16.23	97.66 ± 15.75	102.44 ± 16.36	<0.001
FBG (mg/dL)	106.6 ± 29.9	105.4 ± 26.8	107.7 ± 32.8	0.114
TG (mg/dL)	116.4 ± 61.1	114.7 ± 58.9	118.2 ± 63.2	0.232
HDL (mg/dL)	59.42 ± 16.86	59.60 ± 17.07	59.23 ± 16.65	0.675
LDL (mg/dL)	115.3 ± 35.6	115.0 ± 35.0	115.5 ± 36.2	0.783
TC (mg/dL)	198.0 ± 40.8	197.5 ± 40.1	198.4 ± 41.6	0.676
TyG	8.58 ± 0.60	8.56 ± 0.60	8.60 ± 0.59	0.166
TyG‐BMI	260.4 ± 69.2	250.6 ± 65.5	270.3 ± 71.4	<0.001
TyG‐WC	861.1 ± 166.9	838.9 ± 164.0	883.4 ± 167.0	<0.001
TyG‐WHtR	5.39 ± 1.05	5.25 ± 1.04	5.54 ± 1.04	<0.001
Blood urea nitrogen (mg/dL)	13.67 ± 6.62	13.37 ± 6.21	13.97 ± 7.00	0.066
Blood creatinine (mg/dL)	0.81 ± 0.39	0.80 ± 0.36	0.81 ± 0.42	0.545
Uric acid (mg/dL)	5.08 ± 1.35	5.08 ± 1.36	5.08 ± 1.34	0.938

For categorical variables, P values were analysed by chi‐square tests. For continuous variables, the t‐test was used.

Abbreviations: PSM, propensity score matching; BMI, body mass index; WC, waist circumference; FBG, fast blood glucose; TG, triglyceride; HDL, high‐density lipoprotein; LDL, low‐density lipoprotein; TC, total cholesterol; TyG, triglyceride‐glucose; TyG‐BMI, triglyceride‐glucose‐body mass index; TyG‐WC, triglyceride‐glucose‐waist circumference; TyG‐WHtR, triglyceride‐glucose‐waist‐to‐height ratio.

**TABLE 2:** Cox regression analysis of the relationship between overactive bladder and all‐cause and cardiovascular mortality.

Characteristics	Before PSM		After PSM	
	HR (95% CI)	*P* value	HRR (95% CI)	*P* value
All‐cause mortality				
OAB				
No	Reference		Reference	
Yes	1.414 (1.167–1.714)	<0.001	1.457 (1.107–1.918)	0.007
Cardiovascular mortality				
OAB				
No	Reference		Reference	
Yes	1.528 (1.026–2.274)	0.037	1.939 (1.048–3.589)	0.035

Adjusted covariates: age, race, education level, marital status, hypertension, diabetes, alcohol consumption, smoking status, physical activity status, renal function and blood lipid levels.

Abbreviations: PSM, propensity score matching; CI: confidence interval; aHR, adjusted hazard ratio.

### 3.3 Association between TyG‐related indices and OAB

**FIGURE 3:**
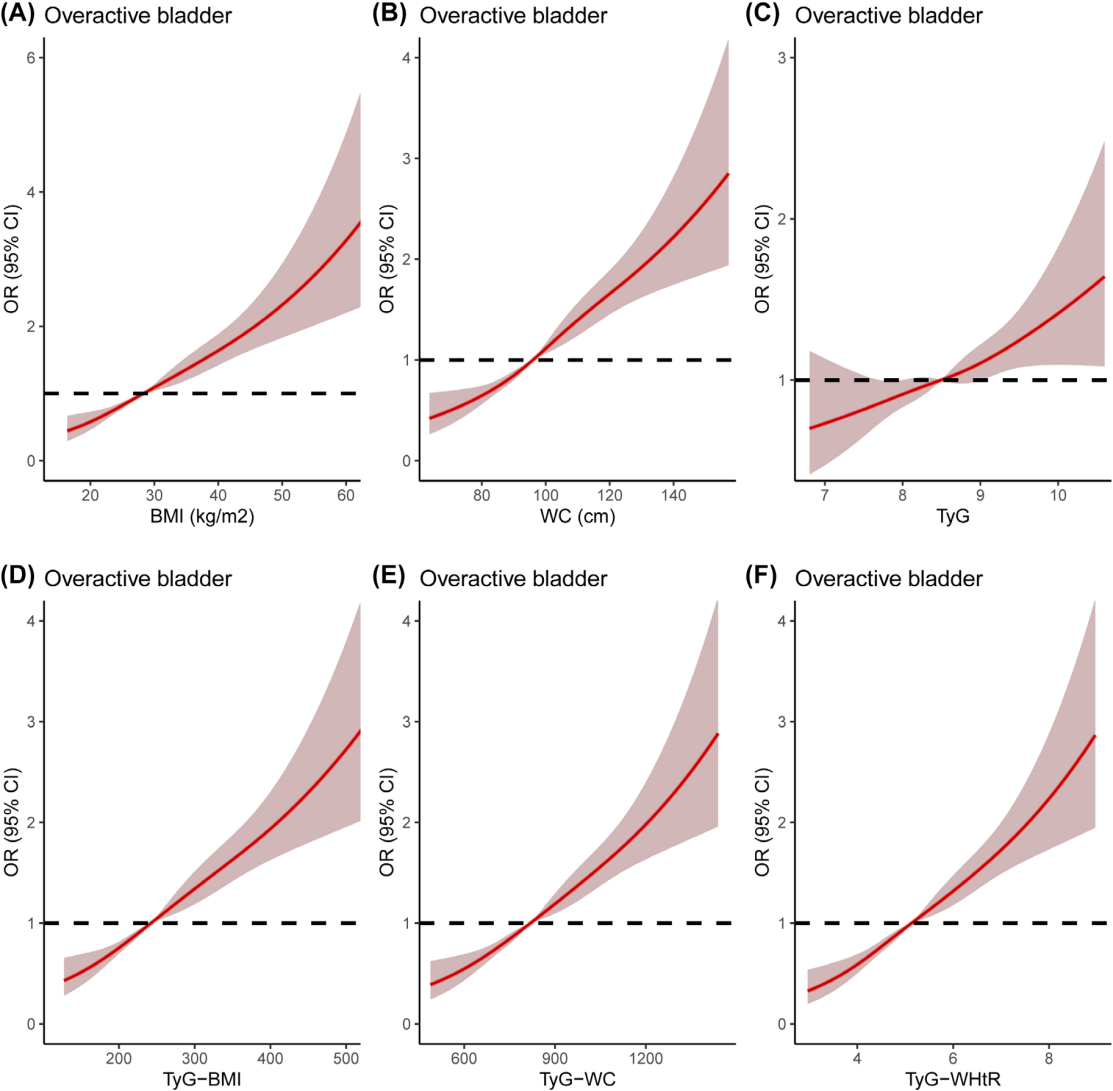
**The dose–response analysis between TyG‐related indices and overactive bladder occurrence before propensity score matching (PSM).** (A) BMI; (B) WC; (C) TyG; (D) TyG‐BMI; (E) TyG‐WC; (F) TyG‐WHtR. SD = standard deviation; CI = confidence interval.

RCS curves showed a nonlinear positive association between OAB and TyG‐related indices, including BMI, WC, TyG, TyG‐BMI, TyG‐WC and TyG‐WHtR before and after PSM (Figure 3 and **Figure** **S3**). ROC curves were further used to assess the accuracy of BMI, WC, TyG, TyG‐BMI, TyG‐WC and TyG‐WHtR in predicting OAB. The results indicated that TyG‐WHtR exhibited the strongest association with OAB among the TyG‐related indices before and after PSM. (**Table** **S5**).

Logistic regression analysis was then used to assess the OR between TyG‐related indices and OAB. The results indicated that BMI, WC, TyG‐BMI, TyG‐WC and TyG‐WHtR were all associated with the risk of OAB after adjusting for all confounding variables. Among these, TyG‐WHtR exhibited the strongest positive association with OAB. Compared to the Q1 of the TyG‐WHtR, the OAB risks for Q2, Q3 and Q4 were 1.564 (95% CI 1.261–1.940, p < 0.001), 2.084 (95% CI 1.663–2.613, p < 0.001) and 3.135 (95% CI 2.450–4.011, p < 0.001), respectively, before PSM (**Figure** **S4**), and 1.644 (95% CI 1.200–2.251, p = 0.002), 2.036 (95% CI 1.472–2.816, p < 0.001) and 2.883 (95% CI 2.084–3.988, p < 0.001), respectively, after PSM (**Figure** **S5**).

## 4 DISCUSSION

In this nationally representative cross‐sectional study, we found that all‐cause mortality and cardiovascular mortality were significantly higher in women with OAB compared to those without OAB. Multivariable Cox regression analysis further revealed that OAB was an independent risk factor for both all‐cause and cardiovascular mortality in women. Interestingly, TyG‐related indices, including BMI, WC, TyG, TyG‐BMI, TyG‐WC and TyG‐WHtR, exhibited a dose‐dependent, nonlinear positive association with OAB. Moreover, ROC curves demonstrated that TyG‐WHtR shows the strongest association with OAB, and multivariate logistic regression analysis indicated that TyG‐WHtR is an independent risk factor for OAB. The positive association between OAB and mortality, as well as the positive association between OAB and TyG‐related indices, were all confirmed in a 1:1 matched cohort after PSM. These findings highlight the importance of OAB as a risk factor for mortality in women and suggest the value of TyG‐related indices as simple and effective biomarkers for evaluating OAB, thereby promoting individualized surveillance and management.

OAB is a common disease in women, with its prevalence increasing with age. OAB can lead to a range of physical, psychological and social problems, including sleep disruption, increased risk of falls, reduced social activity and depression.^21^ Studies have shown that nocturia, a common symptom of OAB, serves as a significant contributor to sleep disruption in adults, severely impairing patients' sleep and overall quality of life, and potentially leading to increased mortality.^22^ Furthermore, Gong et al.^23^ demonstrated that OAB exhibits positive correlations with both the incidence of suicidal ideation among participants experiencing such thoughts and all‐cause mortality. These studies revealed that depression acts as a mediating factor in the association between OAB and suicidal ideation. OAB and urinary incontinence are also strongly associated with cardiovascular diseases like heart failure, contributing to cardiovascular mortality.^24^ Due to frequent urination and urgency, patients with OAB are more likely to have reduced social engagement.^25^

As mentioned earlier, metabolic syndrome is strongly associated with OAB.^26^ A follow‐up study of women over 40 years old in the UK found that diabetes was an independent risk factor for OAB.^27^ A survey involving 5204 women aged 18 and older revealed that BMI was an independent risk factor for OAB,^2^ with women having a BMI greater than 30 being 2.2 times more likely to develop OAB compared to those with a BMI less than 24. In a survey of 518 women with type 2 diabetes in Taiwan,^28^ OAB was significantly more prevalent in women with metabolic syndrome, experiencing more severe symptoms of urgency and nocturia.

TyG index was an effective novel biomarker for assessing insulin resistance (IR), aiding clinicians in their decision‐making.^11^ Studies have shown that an elevated TyG index is significantly associated with an increased risk of type 2 diabetes, making it a valuable tool for diabetes risk screening.^29^, ^30^ Additionally, a higher TyG index is closely linked to an increased risk of diabetes complications, such as atherosclerosis, coronary artery disease, diabetic nephropathy and diabetic retinopathy.^31^ Furthermore, an elevated TyG index is associated with a higher risk of major adverse cardiac events in patients with triple‐vessel disease (TVD).^32^

Obesity is well known to be associated with OAB and urinary incontinence, particularly in women, where the risk of OAB increases with WC, hip circumference and BMI.^33^ Central obesity, defined by WC and WHtR, is a key indicator for assessing the risk of various obesity‐related chronic diseases.^34^ Recent studies have examined new TyG‐related indices that combine the TyG index with obesity indicators, such as TyG‐WC, TyG‐BMI and TyG‐WHtR.^35^ Compared to the original TyG index, these new indices may offer better value for assessing disease outcomes.^36^ WHtR serves as a practical marker for both generalized and abdominal adiposity. Evidence indicates that WHtR demonstrates better accuracy than WC and BMI in identifying cardiometabolic risk factors.^37^ The TyG‐WHtR index, which standardizes adiposity distribution assessment across individuals of varying heights using waist‐to‐height ratio, has been consistently shown in multiple studies to exhibit enhanced value for assessing disease risks.^38^, ^39^ In the current study, we found that TyG‐WHtR outperformed the other indices in assessing the occurrence of OAB, both before and after PSM. Our studies also confirmed that the TyG‐WHtR index has a superior value for assessing cardiovascular disease and related mortality risk compared to the TyG index.^40^ For female OAB patients, regular monitoring of the TyG‐WHtR index may benefit the management of the OAB occurrence. For individuals with high TyG‐WHtR, timely management of blood glucose, triglycerides and waist circumference can potentially reduce the incidence of OAB.

This study is the first comprehensive assessment of the association between OAB and all‐cause and cardiovascular mortality in women, as well as the first evaluation of the association of TyG‐related indices with OAB. The large‐scale population‐based study, combined with a long follow‐up period, enhances the reliability of the data. However, there are several limitations to this study. First, as a retrospective study, the findings need further validation with prospective studies. Second, the diagnosis of OAB was based on OABSS scores, which may be subject to biases, including participant recall bias. NHANES lacks specific data on other comorbidities that may compound the association between overactive bladder and all‐cause mortality. Although the PSM method was used in this study, PSM does not eliminate all potential confounding factors such as lifestyle differences and medication use history. Finally, since the NHANES samples are limited to the US population, the generalizability of the findings to other countries remains unvalidated, highlighting the need for prospective international multicenter studies.

## 5 CONCLUSION

In conclusion, our study demonstrates that OAB is an independent risk factor for both all‐cause mortality and cardiovascular mortality in the female population. Furthermore, the TyG‐WHtR index exhibits the strongest association with OAB, making it a simple and easily calculated clinical biomarker for OAB management.

## AUTHOR CONTRIBUTIONS

Weipu Mao and Sagar Barge: data analysis and writing; Weipu Mao: data collection; Zhaobo Luo: interpretation of the results and revision; WY: interpretation, revision, review and final approval. All authors reviewed the manuscript.

## CONFLICT OF INTEREST STATEMENT

We declare no conflicts of interest.

## ETHICS APPROVAL AND CONSENT TO PARTICIPATE

NHANES was conducted with approval by the National Center for Health Statistics Ethics Review Board and obtained informed written consent from all the individuals involved in the study.

## CONSENT FOR PUBLICATION

Not applicable.

## Supporting information




Figure S1. All‐cause and cardiovascular mortality curves between the overactive and non‐overactive bladder groups before 1:1 propensity score matching (PSM).
Figure S2. Forest plots of different models of multivariate Cox regression analysis of the relation between OAB and all‐cause and cardiovascular deaths in women before 1:1 propensity score matching (PSM).
Figure S3. The dose–response analysis between TyG‐related indices and overactive bladder occurrence after propensity score matching (PSM).
Figure S4. Forest plots of different models of multivariate logistic regression analysis of the relation between TyG‐related indices and overactive bladder occurrence before 1:1 propensity score matching (PSM).
Figure S5. Forest plots of different models of multivariate logistic regression analysis of the relation between TyG‐related indices and overactive bladder occurrence after 1:1 propensity score matching (PSM).
**Table S1**. Conversion of symptom frequencies recorded in NHANES to OABSS scores.
**Table S2**. Baseline characteristics of participants between 2007 and 2018 before PSM.
**Table S3**. Cox regression analysed the relationship between overactive bladder and all‐cause and cardiovascular mortality before PSM.
**Table S4**. Cox regression analysed the relationship between overactive bladder and all‐cause and cardiovascular mortality after PSM.
**Table S5**. Comparison of area under the curve (AUC) between different triglyceride glucose‐related indicators and the presence of overactive bladder before and after PSM.

## Data Availability

The datasets used and/or analysed in this study are publicly available or can be obtained from the corresponding author upon reasonable request.
