# Supplementary material for: Association of overactive bladder with all‐cause and cardiovascular mortality in women: A propensity‐matched NHANES study
Source: BJUI Compass. 2025 Apr 29;6(5):e70022. doi: 10.1002/bco2.70022 (PMC12040721; doi:10.1002/bco2.70022)
Supplement: Supplementary file 1 — Figure S1. All‐cause and cardiovascular mortality curves between the overactive and non‐overactive bladder groups before 1:1 propensity score matching (PSM). Figure S2. Forest plots of different models of multivariate Cox regression analysis of the relation between OAB and all‐cause and cardiovascular deaths in women before 1:1 propensity score matching (PSM). Figure S3. The dose–response analysis between TyG‐related indices and overactive bladder occurrence after propensity score matching (PSM). Figure S4. Forest plots of different models of multivariate logistic regression analysis of the relation between TyG‐related indices and overactive bladder occurrence before 1:1 propensity score matching (PSM). Figure S5. Forest plots of different models of multivariate logistic regression analysis of the relation between TyG‐related indices and overactive bladder occurrence after 1:1 propensity score matching (PSM). Table S1. Conversion of symptom frequencies recorded in NHANES to OABSS scores. Table S2. Baseline characteristics of participants between 2007 and 2018 before PSM. Table S3. Cox regression analysed the relationship between overactive bladder and all‐cause and cardiovascular mortality before PSM. Table S4. Cox regression analysed the relationship between overactive bladder and all‐cause and cardiovascular mortality after PSM. Table S5. Comparison of area under the curve (AUC) between different triglyceride glucose‐related indicators and the presence of overactive bladder before and after PSM. [file BCO2-6-e70022-s001.docx]

**Association of overactive bladder with all-cause and** **cardiovascular mortality in women: a propensity matched NHANES study**

Weipu Mao^1#^, Sagar Barge^1#^, Zhaobo Luo^1^, Weiqun Yu^1^

**Author Affiliations:**

^1^Department of Medicine, Beth Israel Deaconess Medical Center, Harvard Medical School, Boston, Massachusetts, USA

^*^**Corresponding Author:**

Weiqun Yu, Department of Medicine, Beth Israel Deaconess Medical Center, RN380B, 99 Brookline Avenue, Boston, MA 02215, USA.

Email: wyu2@bidmc.harvard.edu

^#^**Contributed equally**

**Running head: OAB and TyG-related Index**

**Figure legends**

**
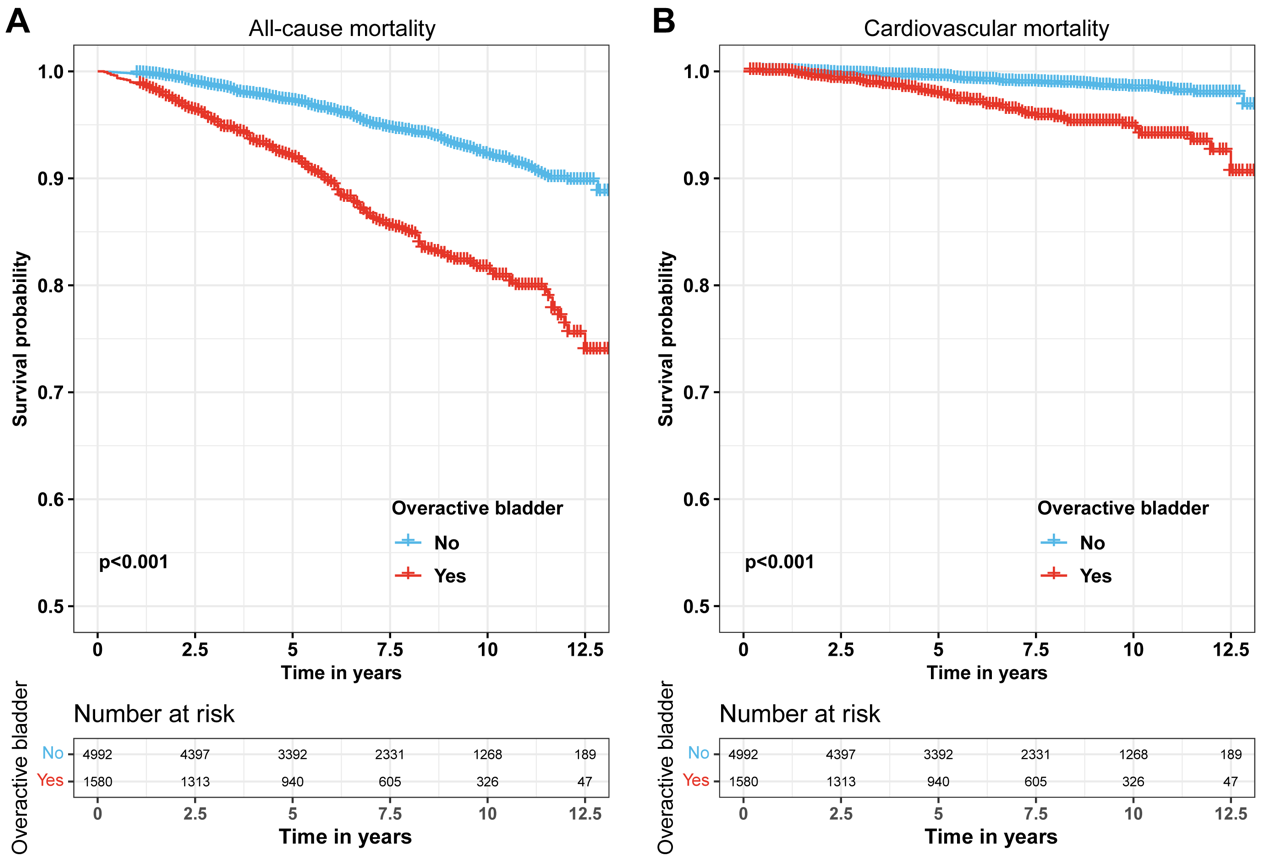
**

**Figure S1.** **All-cause and cardiovascular mortality curves between the overactive and non-overactive bladder groups before 1:1 propensity score matching (PSM).**

(A) All-cause mortality curves; (B) Cardiovascular mortality curves.


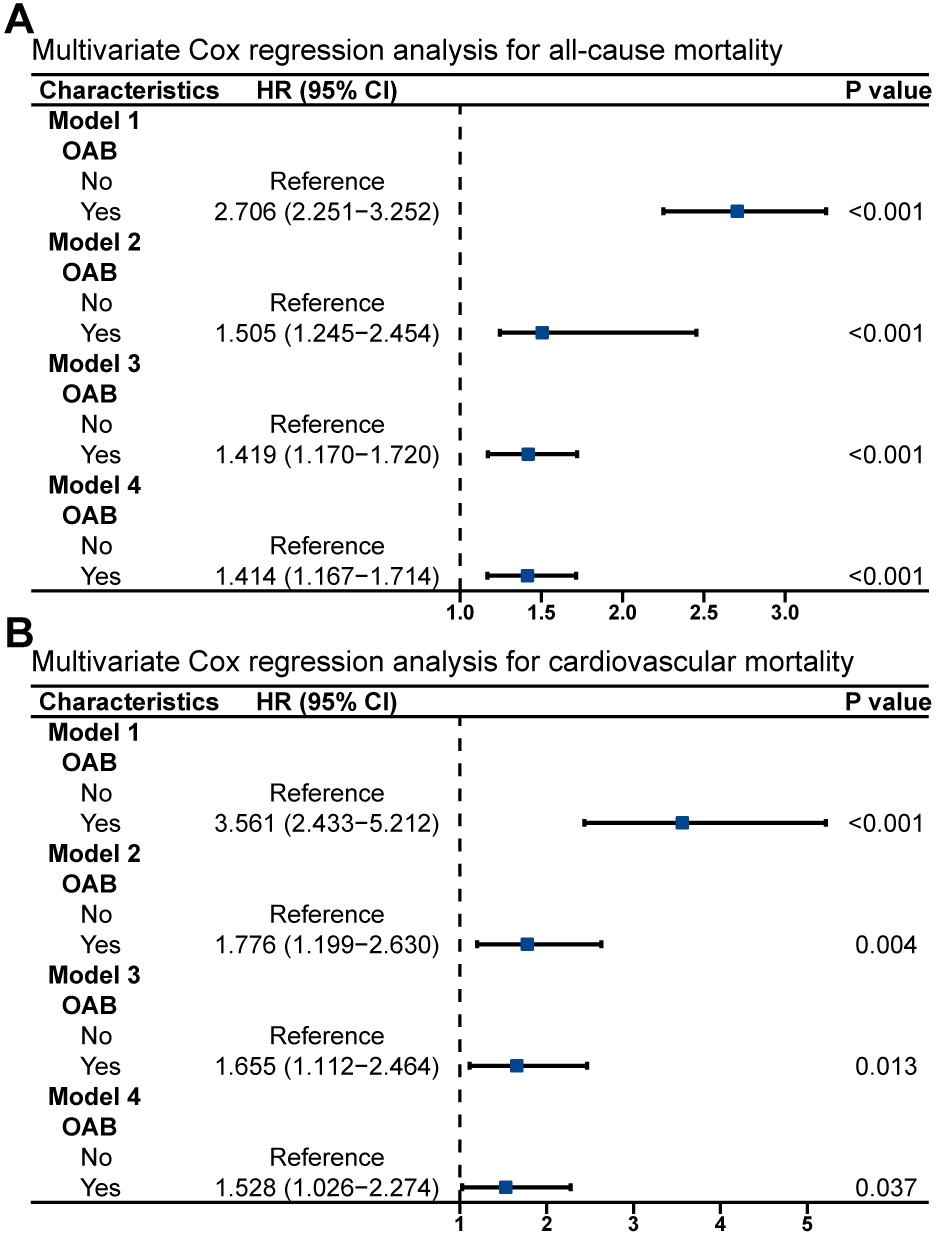


**Figure S2.** **Forest plots of different models of multivariate Cox regression analysis of the relation between OAB and all-cause and cardiovascular deaths in women before 1:1 propensity score matching (PSM).**

**
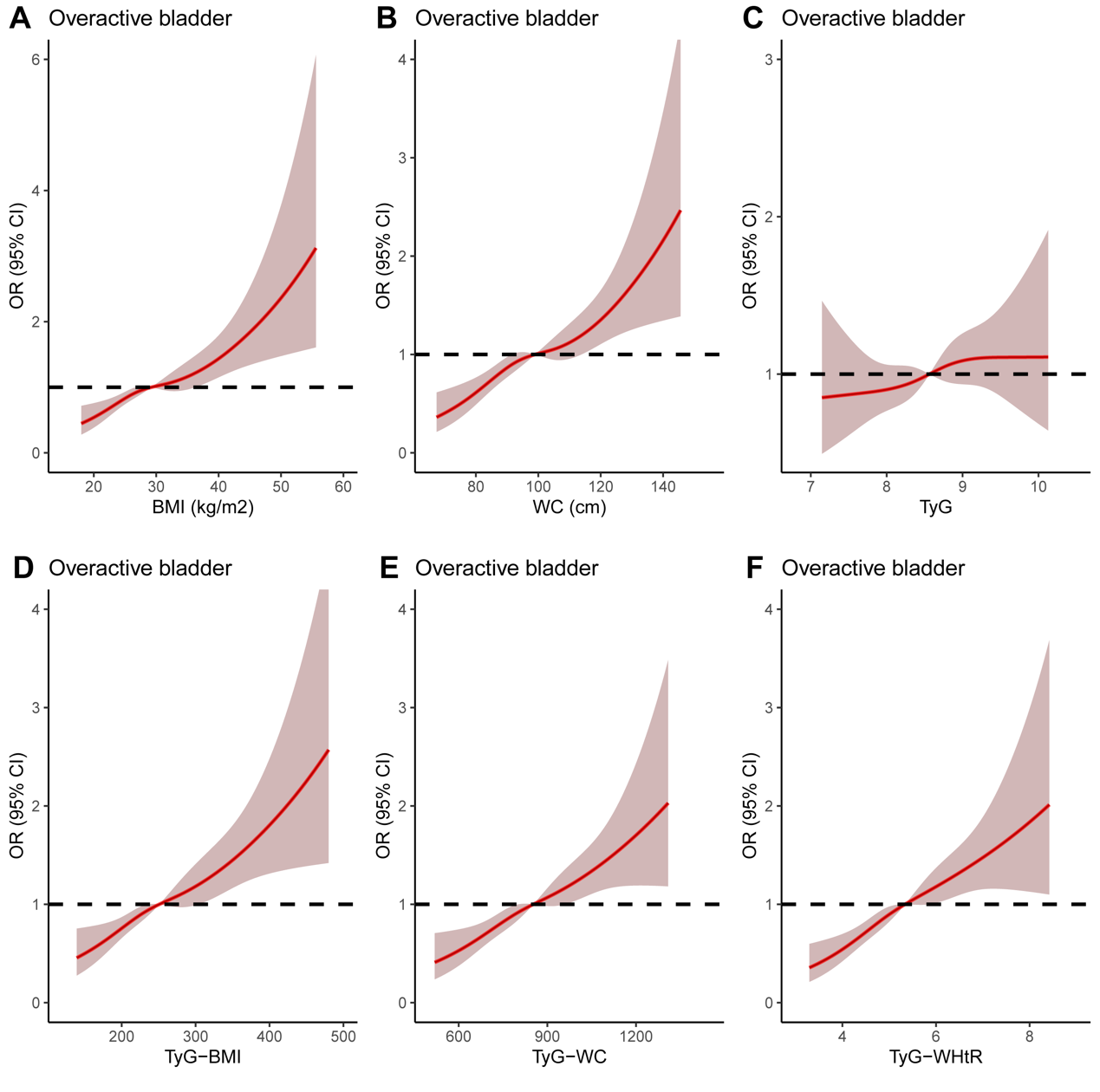
**

(A) All-cause mortality; (B) Cardiovascular mortality.

**Figure S3. The dose-response analysis between TyG-related indices and overactive bladder occurrence after propensity score matching (PSM).**

(A) BMI; (B) WC; (C) TyG; (D) TyG-BMI; (E) TyG-WC; (F) TyG-WHtR.

SD = standard deviation; CI = confidence interval.


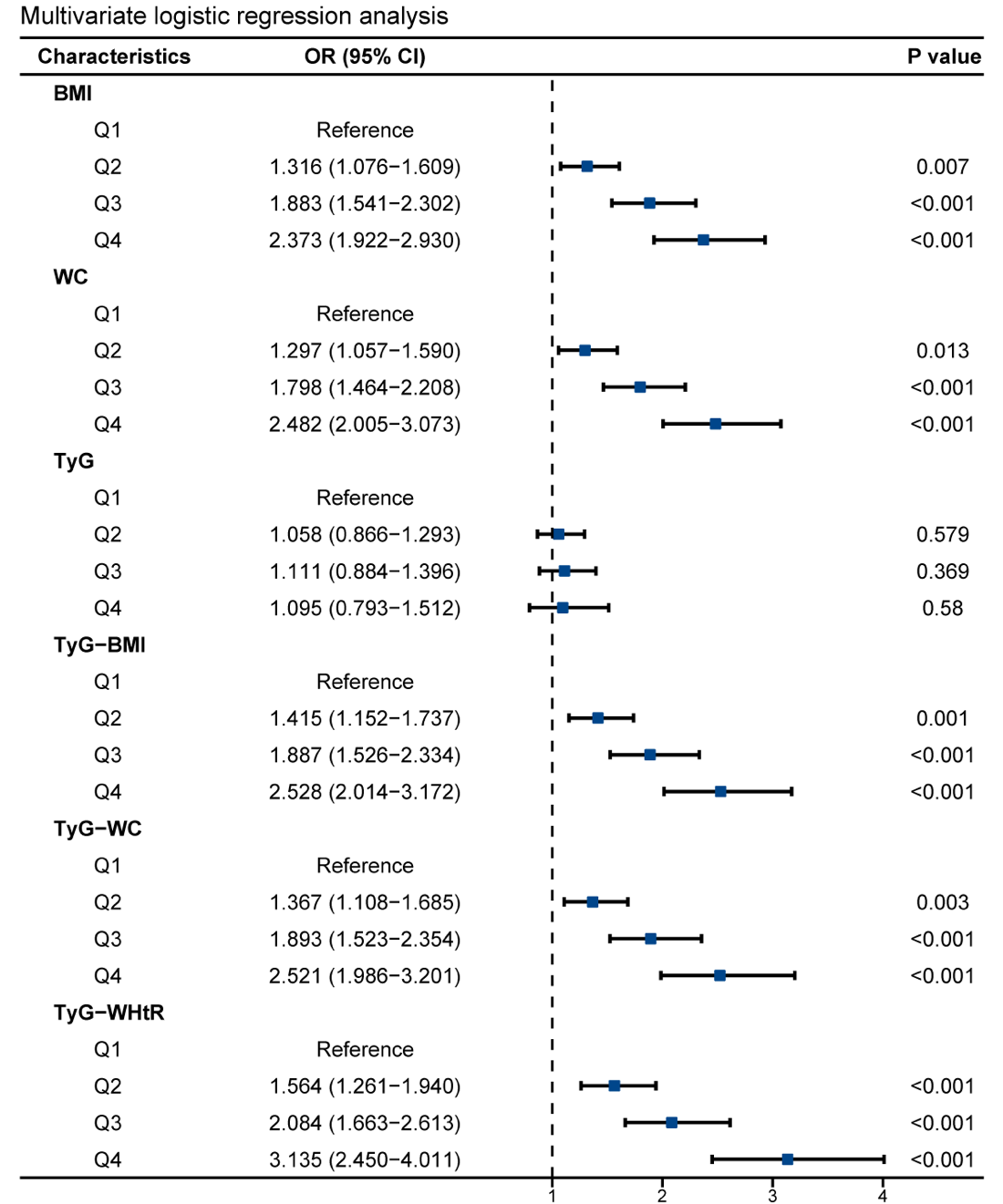


**Figure S4. Forest plots of different models of multivariate logistic regression analysis of the relation between TyG-related indices and overactive bladder occurrence before 1:1 propensity score matching (PSM).**

**
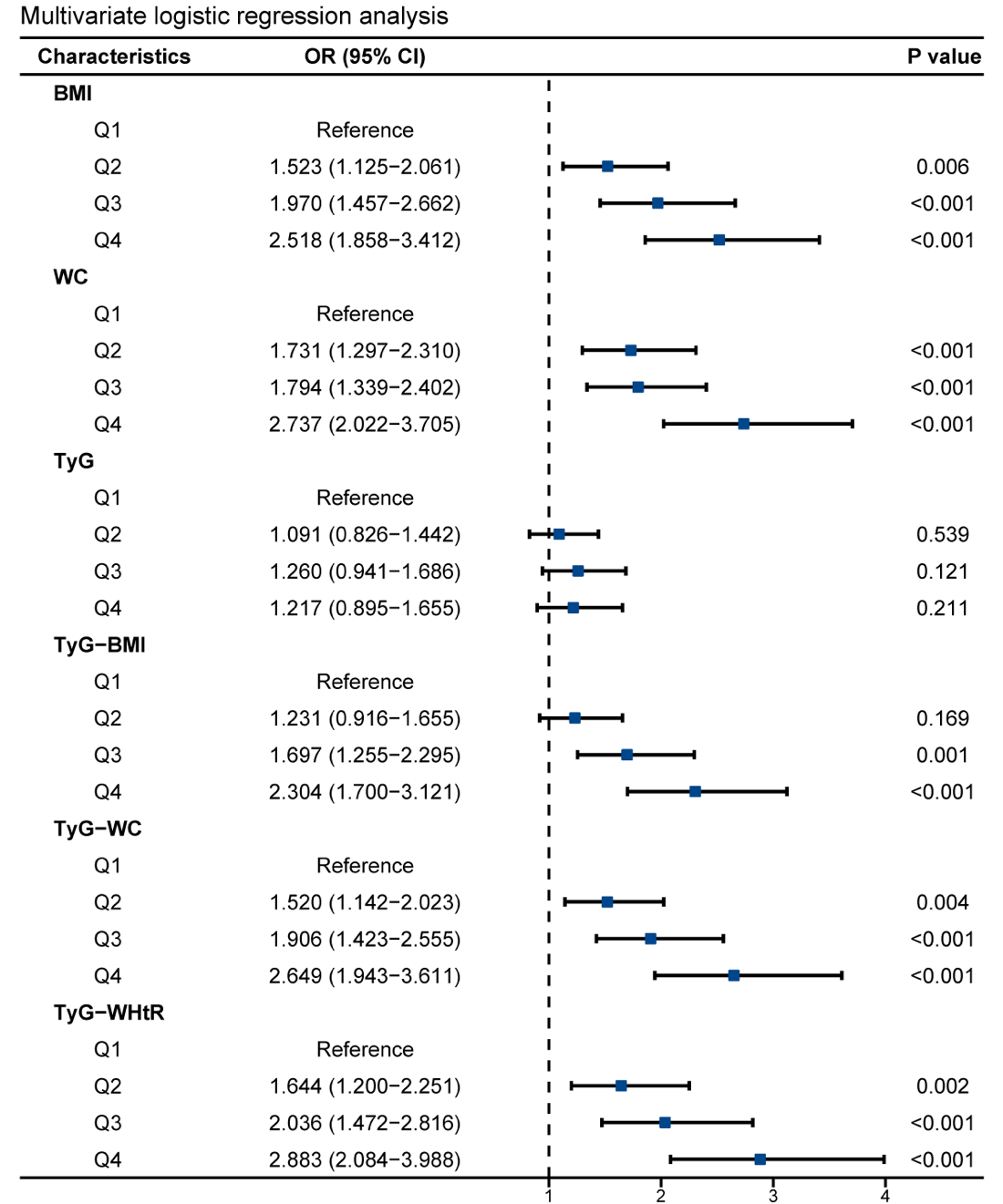
**

**Figure S5. Forest plots of different models of multivariate logistic regression analysis of the relation between TyG-related indices and overactive bladder occurrence after 1:1 propensity score matching (PSM).**

**Table S1**. Conversion of symptom frequencies recorded in NHANES to OABSS scores.

| **NHANES score** | **OABSS score** |
| --- | --- |
| Urge urinary incontinence frequency | Urge urinary incontinence score |
| Never | 0 |
| Less than once a month | 1 |
| A few times a month | 1 |
| A few times a week | 2 |
| Every day and/or night | 3 |
| Nocturia frequency Nocturia score | Nocturia frequency Nocturia score |
| 0 | 0 |
| 1 | 1 |
| 2 | 2 |
| 3 | 3 |
| 4 | 3 |
| 5 or more | 4 |

Abbreviations: NHANES, National Health and Nutrition Examination Survey; OABSS, Overactive Bladder Symptom Score

**Table S2**. Baseline characteristics of participants between 2007 and 2018 before PSM.

| **Characteristic** | **Total** | **None-overactive bladder** | **Overactive bladder** | **P**  **value** |
| --- | --- | --- | --- | --- |
|  | **No. (%)** | **No. (%)** | **No. (%)** |  |
| Total patients | 6580 | 4999 (76.0) | 1581 (24.0) |  |
| Age, years |  |  |  | <0.001 |
| <40 | 2126 (32.3) | 1868 (37.4) | 258 (16.3) |  |
| 40-60 | 2357 (35.8) | 1820 (36.4) | 537 (34.0) |  |
| >60 | 2097 (31.9) | 1311 (26.2) | 786 (49.7) |  |
| Race |  |  |  | <0.001 |
| Mexican American | 1005 (15.3) | 756 (15.1) | 249 (15.7) |  |
| Other Hispanic | 756 (11.5) | 568 (11.4) | 188 (11.9) |  |
| Non-Hispanic white | 2745 (41.7) | 2146 (42.9) | 599 (37.9) |  |
| Non-Hispanic black | 1342 (20.4) | 914 (18.3) | 428 (27.1) |  |
| Other | 732 (11.1) | 615 (12.3) | 117 (7.4) |  |
| Education level |  |  |  | <0.001 |
| Less than high school | 1514 (23.0) | 987 (19.7) | 527 (33.3) |  |
| High school or equivalent | 1425 (21.7) | 1056 (21.1) | 369 (23.3) |  |
| College or above | 3641 (55.3) | 2956 (59.1) | 685 (43.3) |  |
| Marital status |  |  |  | <0.001 |
| Married | 3071 (46.7) | 2449 (49.0) | 622 (39.3) |  |
| Unmarried | 3509 (53.3) | 2550 (51.0) | 959 (60.7) |  |
| Hypertension |  |  |  | <0.001 |
| Yes | 2449 (37.2) | 1552 (31.0) | 897 (56.7) |  |
| No | 4131 (62.8) | 3447 (69.0) | 684 (43.3) |  |
| Diabetes |  |  |  | <0.001 |
| Yes | 802 (12.2) | 436 (8.7) | 366 (23.1) |  |
| No | 5617 (85.4) | 4451 (89.0) | 1166 (73.8) |  |
| Borderline | 161 (2.4) | 112 (2.2) | 49 (3.1) |  |
| Alcohol consumption |  |  |  | <0.001 |
| Yes | 4306 (65.4) | 3351 (67.0) | 955 (60.4) |  |
| No | 2274 (34.6) | 1648 (33.0) | 626 (39.6) |  |
| Smoking status |  |  |  | <0.001 |
| Never | 4230 (64.3) | 3275 (65.5) | 955 (60.4) |  |
| Former | 1247 (19.0) | 900 (18.0) | 347 (21.9) |  |
| Current | 1103 (16.8) | 824 (16.5) | 279 (17.6) |  |
| Vigorous work activity |  |  |  | 0.926 |
| Yes | 816 (12.4) | 621 (12.4) | 195 (12.3) |  |
| No | 5764 (87.6) | 4378 (87.6) | 1386 (87.7) |  |
| Moderate work activity |  |  |  | 0.001 |
| Yes | 2182 (33.2) | 1713 (34.3) | 469 (29.7) |  |
| No | 4398 (66.8) | 3286 (65.7) | 1112 (70.3) |  |
| Vigorous recreational activity |  |  |  | <0.001 |
| Yes | 1132 (17.2) | 1023 (20.5) | 109 (6.9) |  |
| No | 5448 (82.8) | 3976 (79.5) | 1472 (93.1) |  |
| Moderate recreational activity |  |  |  | <0.001 |
| Yes | 2685 (40.8) | 2195 (43.9) | 490 (31.0) |  |
| No | 3895 (59.2) | 2804 (56.1) | 1091 (69.0) |  |
| BMI (kg/m^2^) | 29.57±7.51 | 28.78±7.18 | 32.04±8.00 | <0.001 |
| WC (cm) | 97.77±16.79 | 95.74±16.29 | 104.19±16.77 | <0.001 |
| FBG (mg/dL) | 106.8±33.8 | 103.75±29.14 | 116.3±44.0 | <0.001 |
| TG (mg/dL) | 110.5±61.2 | 106.66±59.52 | 122.8±64.6 | <0.001 |
| HDL (mg/dL) | 58.78±16.19 | 59.13±16.06 | 57.68±16.54 | 0.002 |
| LDL (mg/dL) | 113.9±35.6 | 113.5±35.1 | 115.1±37.4 | 0.111 |
| TC (mg/dL) | 194.8±41.0 | 194.0±40.5 | 197.4±42.3 | 0.004 |
| TyG | 8.51±0.63 | 8.45±0.62 | 8.70±0.63 | <0.001 |
| TyG-BMI | 253.0±71.6 | 244.6±68.5 | 279.4±74.8 | <0.001 |
| TyG-WC | 836.0±175.4 | 813.0±170.0 | 908.9±173.7 | <0.001 |
| TyG-WHtR | 5.22±1.11 | 5.06±1.07 | 5.70±1.09 | <0.001 |
| Blood urea nitrogen (mg/dL) | 12.94±5.92 | 12.49±5.44 | 14.36±7.04 | <0.001 |
| Blood creatinine (mg/dL) | 0.77±0.30 | 0.75±0.28 | 0.82±0.37 | <0.001 |
| Uric acid (mg/dL) | 4.93±1.30 | 4.84±1.25 | 5.24±1.42 | <0.001 |

For categorical variables, P values were analyzed by chi-square tests. For continuous variables, the t-test was used.

Abbreviations: PSM, propensity score matching; BMI, body mass index; WC, waist circumference; FBG, fast blood glucose; TG, triglyceride; HDL, high-density lipoprotein; LDL, low-density lipoprotein; TC, total cholesterol; TyG, triglyceride-glucose; TyG-BMI, triglyceride-glucose-body mass index; TyG-WC, triglyceride-glucose-waist circumference; TyG-WHtR, triglyceride-glucose-waist-to-height ratio.

**Table S3**. Cox regression analysed the relationship between overactive bladder and all-cause and cardiovascular mortality before PSM.

| **Characteristics** | **Model 1** | | **Model 2** | | **Model 3** | | **Model 4** | |
| --- | --- | --- | --- | --- | --- | --- | --- | --- |
|  | **aHR (95% CI)** | **P value** | **aHR (95% CI)** | **P value** | **aHR (95% CI)** | **P value** | **aHR (95% CI)** | **P value** |
| All-cause mortality |  |  |  |  |  |  |  |  |
| OAB |  |  |  |  |  |  |  |  |
| No | Reference |  | Reference |  | Reference |  | Reference |  |
| Yes | 2.706 (2.251-3.252) | <0.001 | 1.505 (1.245-2.454) | <0.001 | 1.419 (1.170-1.720) | <0.001 | 1.414 (1.167-1.714) | <0.001 |
| Cardiovascular mortality |  |  |  |  |  |  |  |  |
| OAB |  |  |  |  |  |  |  |  |
| No | Reference |  | Reference |  | Reference |  | Reference |  |
| Yes | 3.561 (2.433-5.212) | <0.001 | 1.776 (1.199-2.630) | 0.004 | 1.655 (1.112-2.464) | 0.013 | 1.528 (1.026-2.274) | 0.037 |

Adjusted covariates: model 1: univariate analysis; model 2: age, race, education level, and marital status; model 3: model 2 plus hypertension, diabetes, alcohol consumption and smoking status; model 4: model 3 plus physical activity status, renal function, and blood lipid levels.

Abbreviations: PSM, propensity score matching; CI: confidence interval; aHR, adjusted hazard ratio.

**Table S4**. Cox regression analysed the relationship between overactive bladder and all-cause and cardiovascular mortality after PSM.

| **Characteristics** | **Model 1** | | **Model 2** | | **Model 3** | | **Model 4** | |
| --- | --- | --- | --- | --- | --- | --- | --- | --- |
|  | **aHR (95% CI)** | **P value** | **aHR (95% CI)** | **P value** | **aHR (95% CI)** | **P value** | **aHR (95% CI)** | **P value** |
| All-cause mortality |  |  |  |  |  |  |  |  |
| OAB |  |  |  |  |  |  |  |  |
| No | Reference |  | Reference |  | Reference |  | Reference |  |
| Yes | 1.448 (1.103-1.899) | 0.008 | 1.458 (1.111-1.914) | 0.007 | 1.491 (1.136-1.957) | 0.004 | 1.457 (1.107-1.918) | 0.007 |
| Cardiovascular mortality |  |  |  |  |  |  |  |  |
| OAB |  |  |  |  |  |  |  |  |
| No | Reference |  | Reference |  | Reference |  | Reference |  |
| Yes | 2.110 (1.142-3.898) | 0.017 | 2.182 (1.181-4.033) | 0.013 | 2.052 (1.112-3.789) | 0.022 | 1.939 (1.048-3.589) | 0.035 |

Adjusted covariates: model 1: univariate analysis; model 2: age, race, education level, and marital status; model 3: model 2 plus hypertension, diabetes, alcohol consumption and smoking status; model 4: model 3 plus physical activity status, renal function, and blood lipid levels.

Abbreviations: PSM, propensity score matching; CI: confidence interval; aHR, adjusted hazard ratio.

**Table S5**. Comparison of area under the curve (AUC) between different triglyceride glucose-related indicators and the presence of overactive bladder before and after PSM.

| **Characteristics** | **Before PSM** | |  | **After PSM** | |
| --- | --- | --- | --- | --- | --- |
|  | **AUC** | **95% CI** |  | **AUC** | **95% CI** |
| BMI | 0.628 | 0.612-0.643 |  | 0.581 | 0.554-0.608 |
| WC | 0.648 | 0.633-0.663 |  | 0.582 | 0.555-0.609 |
| TyG | 0.610 | 0.594-0.626 |  | 0.519 | 0.491-0.546 |
| TyG-BMI | 0.643 | 0.628-0.658 |  | 0.580 | 0.553-0.607 |
| TyG-WC | 0.659 | 0.644-0.674 |  | 0.576 | 0.549-0.603 |
| TyG-WHtR | 0.668 | 0.653-0.683 |  | 0.581 | 0.554-0.608 |

Abbreviations: AUC, area under the curve; PSM, propensity score matching; CI: confidence interval; BMI, body mass index; WC, waist circumference; TyG, triglyceride-glucose; TyG-BMI, triglyceride-glucose-body mass index; TyG-WC, triglyceride-glucose-waist circumference; TyG-WHtR, triglyceride-glucose-waist-to-height ratio.
